# CONTEXT-DEPENDENT SEXUAL CHANGES DURING WOMEN’S MIDLIFE TRANSITIONS

**DOI:** 10.1093/geroni/igac059.1921

**Published:** 2022-12-20

**Authors:** Amber Watts, Sarah Jen

**Affiliations:** University of Kansas, Lawrence, Kansas, United States; University of Kansas, Lawrence, Kansas, United States

## Abstract

For women, midlife represents an important stage of transition, including shifts in physiological, social, and sexual experiences. Prior research demonstrates that women’s sexuality is more dynamic and context-dependent than men’s. Most research focused on women’s sexuality in mid- to later-life emphasizes physiological changes, while largely ignoring changes stemming from social, psychological, and relational contexts. The present study examined midlife women’s diverse sexual experiences within the context of their lives. We conducted semi-structured interviews with 27 women, ages 39-57 and used interpretive phenomenological analysis to investigate perceptions and interpretations of midlife sexual experiences and changes. Themes included changes in sexual engagement, unwanted sexual experiences, body image, and sexual healthcare. Participants reported changes in frequency of sex and sexual desire within the context of their diverse social roles and identities, prior intimate relationships, and sexual health. Women contrasted perceptions of their own bodies with societal perceptions of sexiness. Frequently reported negative experiences with sexual healthcare informed a distrust of healthcare systems. The diverse and changing nature of participants’ experiences supports prior evidence of sexual fluidity and context-dependence. By questioning societal expectations around sexuality and body image, participants illustrated the potential of counternarratives to combat dominant beliefs and stereotypes about midlife women’s sexuality. To improve sexual health and education, psychoeducational interventions and improved training for healthcare professionals are needed.

